# Dosimetric Evaluation Between the Conventional Volumetrically Modulated Arc Therapy (VMAT) Total Body Irradiation (TBI) and the Novel Simultaneous Integrated Total Marrow Approach (SIMBa) VMAT TBI

**DOI:** 10.7759/cureus.15646

**Published:** 2021-06-14

**Authors:** Dennis Stanley, Kristen McConnell, Zohaib Iqbal, Ashlyn Everett, Jonathan Dodson, Kimberly Keene, Andrew McDonald

**Affiliations:** 1 Radiation Oncology, The University of Alabama at Birmingham, Birmingham, USA; 2 Radiation Oncology/Medical Physics, The University of Alabama at Birmingham, Birmingham, USA

**Keywords:** total body irradiation, tbi, vmat, simba, total marrow

## Abstract

Purpose

The purpose of this study was to assess the treatment planning feasibility of volumetrically modulated arc therapy total body irradiation (VMAT TBI) using a simultaneous integrated marrow and body approach (SIMBa). We also aimed to compare SIMBa TBI with the more conventional VMAT TBI approach using the entire body as the target. The goal of using an integrated approach like SIMBa is to balance the known clinical benefit of TBI with the toxicity decrease of Total Marrow Irradiation (TMI) using two prescription volumes. In anticipation of a clinical trial to investigate a novel conditioning regimen that uses SIMBa, our institution retrospectively analyzed the dosimetric differences between 20 clinical VMAT TBI which were re-planned using SIMBa.

Methods

Twenty patients who previously received conventional VMAT TBI at our institution with a dose of 12 Gy in six fractions were re-planned using SIMBa with a planning aim of delivering a uniform dose of 12 Gy to at least 90% of the PTV_BodyEval. The planning aims of SIMBa were to deliver a uniform dose of 12 Gy to at least 90% of the PTV_Marrow and 8 Gy to at least 90% of the PTV_TotalBody while limiting the mean lung dose to less than 8 Gy. The plans were normalized so that 100% of the PTV_Marrow received at least 90% of the dose with the PTV_TotalBody optimized to stay as close to 100% at 90% as possible.

Results

All 20 patient plans achieved 12 Gy/8 Gy to at least 90% of the PTV_Marrow and PTV_TotalBody, respectively, with max doses of <16 Gy (130%). As compared with the delivered TBI, the following reductions in mean dose were notable: small bowel 21.3±4.2%, lung 16.3±7.9%, heart 25.3±8.6%, and kidney 16.4±6.2%. Coverage of the sanctuary sites was maintained despite a significant reduction to sensitive organs at risk (OARs).

Conclusion

This study supports that VMAT TBI treatment planning with SIMBa is feasible. In this sample, SIMBa provided dosimetrically similar doses to marrow and sanctuary site doses as TBI while achieving lower doses to OARs. A clinical trial is needed to investigate the clinical implications of VMAT TBI with SIMBa.

## Introduction

Total body irradiation (TBI) is a traditional aspect of myeloablative conditioning regimes to eradicate malignant cells [1, 2]. Additionally, when patients are undergoing an allogeneic hematopoietic stem cell transplantation (HCT), TBI provides strong immunosuppression to prevent the rejection of donor hematopoietic cells [1, 2]. For acute lymphoid lymphoma (ALL) and acute myeloid leukemia (AML), HCT is indicated in a variety of scenarios. Radiation-containing regimens in preparation are generally preferred in patients with ALL [1] but HCT with TBI is not limited to ALL/AML alone. The rationale for using radiation in conditioning regimens include its ability to treat “chemotherapy sanctuary sites”, including the central nervous system and testes; deliver relatively homogeneous dose to all tissues without dependence on blood supply, biodistribution, and metabolism; destroy the chemotherapy-refractory colonies of malignant disease; and suppress the immune system in preparation for the engraftment of donor stem cells [1]. Despite its important role in HCT, TBI also is noted to have potentially severe acute complications, including life-threatening pneumonitis. This is mitigated through fractionation; but even then, when administered concurrently with chemotherapy, it still occurs in about 25% of patients [1]. Since the conventional fractionated high-dose TBI involves the irradiation of whole organs, there are associated long-term risks, such as the formation of cataracts in 30-40% of the patients [3], gonadal failure [4], thyroid and kidney dysfunction [5, 6], and decreased bone mineral density [7]. It has also been shown that ALL patients receiving radiation are at an increased risk for the development of cardiometabolic traits as compared to those patients who received only chemotherapy [8].

The setup and planning for TBI deliveries ultimately drive the dose distributions, with traditional TBI treatment methodologies being unable to specifically target anything besides the entire body. The traditional setups include static extended distance treatment fields commonly with the patient standing, but recently, due to equipment and workflow changes, supine multi-isocenter volumetric modulated arc therapy (VMAT) techniques have become increasingly popular [9]. By moving to a VMAT TBI technique, there is an opportunity to target specific tissues, which could reduce overall organ doses, reduce toxicity, and allow for dose escalation to a specific area, such as total marrow irradiation (TMI) [10]. Early TMI studies, done with the tomotherapy platform, were similar in concept and have been studied in the past. There are clinical trials underway with early results indicating it is feasible and comes with comparable mortality rates [1, 10, 11].

Total marrow irradiation can reduce toxicities, but studies have shown that sanctuary sites are not adequately treated [10, 12, 13]. An alternative strategy using VMAT is to perform a low dose of TBI while escalating the dose to the marrow using a simultaneous integrated marrow and body approach (SIMBa). The goal of using an integrated approach like SIMBa is to balance the known clinical benefit of TBI with the toxicity decrease of TMI using two prescription volumes simultaneously integrated and delivered with 6MV VMAT. We performed this study to assess the treatment planning feasibility of VMAT TBI with SIMBa and to quantify radiation exposure to organs at risk as compared to conventional VMAT TBI.

## Materials and methods

Twenty randomly selected patients who previously received conventional VMAT TBI at our institution with a dose of 12 Gy in six fractions were re-planned using the SIMBa technique. For CT simulation, all patients received our standard planning scans, consisting of two planning CT images obtained with the patient in the head‐first supine (HFS) orientation, scanning from the top of the skull to the mid‐thigh, and a feet-first supine (FFS) from the bottom of the feet to the mid-pelvis. The patients were simulated with a thermoplastic mask over the head and neck region and a full-body vacuum bag for immobilization. The arms were placed as close to the sides of the patient as possible to reduce the field size needed in later planning while also reducing any potential air gaps. 

Conventional VMAT TBI planning

Our conventional VMAT TBI plans were generated using Varian Eclipse™ version 15.6 (Varian Medical Systems, Inc., Palo Alto, USA) treatment planning system following a procedure adapted from Ouyang et al. [14]. Isocenters were placed along the patient's longitudinal axis starting at the superior portion head with a total of six isocenters created: Head, Chest, Abdomen, Pelvis, Upper Leg, and Lower Leg. Isocenters were placed to ensure a minimum of 5 cm overlap in the anterior-posterior (AP)-defined field size of each field. For the superior four isocenters (Head, Chest, Abdomen, and Pelvis) one to three 6 MV VMAT arcs were used. For the two most inferior isocenters (Upper Leg and Lower Leg), two to three AP- PA fields were utilized. For the AP/posterior-anterior (PA) beams, the junctions and hotspots were modulated using a standard “field-in-field” MLC sequence technique prior to the optimization of the arc fields. Three planning target volume (PTV) targets were created to facilitate planning: PTV_BodyEval, PTV_Upper, and PTV_Lower. PTV_BodyEval was defined as the Eclipse™-defined body contour with a 5 mm retraction from the skin minus the entire lung volume/any optional OARs and served as the primary target. PTV_Upper represented the portion of the PTV_BodyEval that encompassed the VMAT arc isocenters (Head, Chest, Abdomen, and Pelvis) and the PTV_Lower represented the portion of the PTV_BodyEval that encompassed the Fiel-in-Field AP/PA technique isocenters (Upper Leg and Lower Leg). It should be noted that PTV_Upper + PTV_Lower equals PTV_BodyEval.

To create a base dose plan for optimization, the PTV_Lower was planned and calculated first. Then, the remaining arc fields used to treat PTV_Upper were simultaneously optimized for coverage and organ sparing. Since previous studies [15-21] have shown that lower dose rates can reduce pulmonary complications, the dose rate for the arcs that directly irradiate the lungs was set to 40 MU/min while all other arcs were increased to a dose rate of 300 MU/min, following the guidance of Held et al. [16] to reduce the treatment time.

For our conventional VMAT TBI, the prescription is 12 Gy in six fractions with a planning aim of delivering a uniform dose of 12 Gy to at least 90% of the PTV_BodyEval.

SIMBa planning

**Figure 1:**
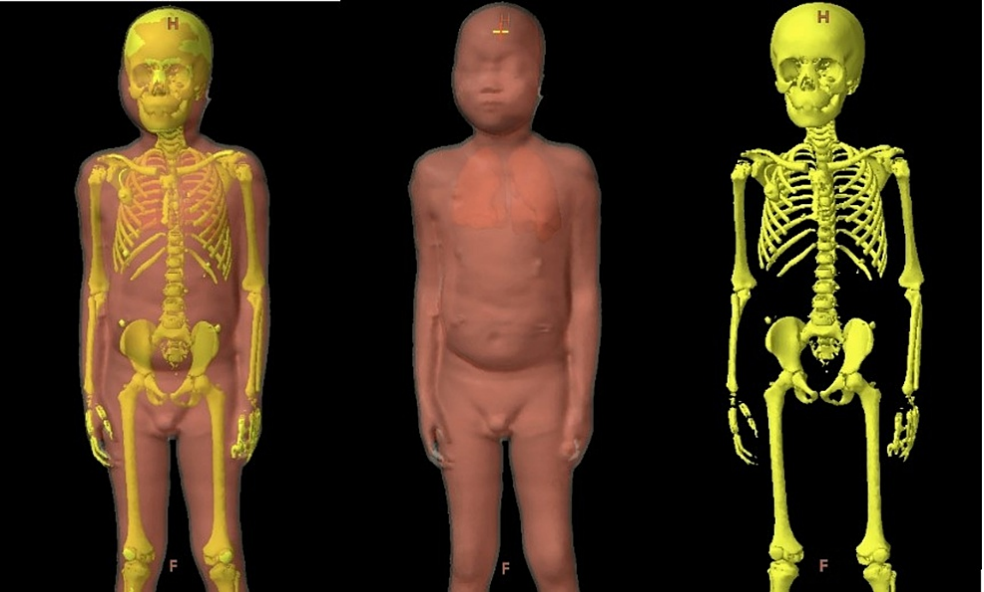
Example targets for a representative patient. (Left) SIMBa targets shown as PTV_TotalBody (8 Gy) and PTV_Marrow (12 Gy), (middle) conventional VMAT TBI target shown as PTV_BodyEval (12 Gy), and (right) PTV_Marrow is shown VMAT: volumetric modulated arc therapy; TBI: total body irradiation; SIMBa: simultaneous integrated marrow and body approach; PTV: planning target volume

To create the retrospective SIMBa plans, the Varian Eclipse™ version 15.6 treatment planning system was used to create two additional planning target volumes: PTV_Marrow and PTV_TotalBody. PTV_Marrow was defined as the entirety of the skeletal system and was consistent with targets found in previous TMI studies [10, 12, 13, 22, 23]. PTV_Marrow was contoured using the auto segmentation technique with bone window level in the Varian Eclipse™ treatment planning system with a minor cleanup. PTV_TotalBody was defined as the PTV_BodyEval minus PTV_Marrow. Table 1 and Figure 1 show the target volumes and their corresponding anatomy for both the conventional VMAT TBI and the SIMBa techniques.

**Table 1:** The target and PTV evaluation volume definitions for conventional and SIMBa VMAT planning VMAT: volumetric modulated arc therapy; SIMBa: simultaneous integrated marrow and body approach; PTV: planning target volume; AP: anterior-posterior; PA: posterior-anterior

	Target definition – Conventional VMAT TBI	Target definition – SIMBa
Body	Automatically generated External contour	
PTV_BodyEval	Automatically generated External contour (Body) with a 5mm uniform retraction away from skin minus the lung volume and any optional OARs	
PTV_Upper	The portion of the PTV_BodyEval that encompassed the VMAT arc isocenters (Head, Chest, Abdomen and Pelvis)	
PTV_Lower	The portion of the PTV_BodyEval that encompassed the field-in-field AP/PA technique isocenters (Upper Leg and Lower Leg).	
PTV_Marrow	-	Contoured skeletal system
PTV_TotalBody	-	PTV_BodyEval minus the PTV_Marrow

**Figure 2:**
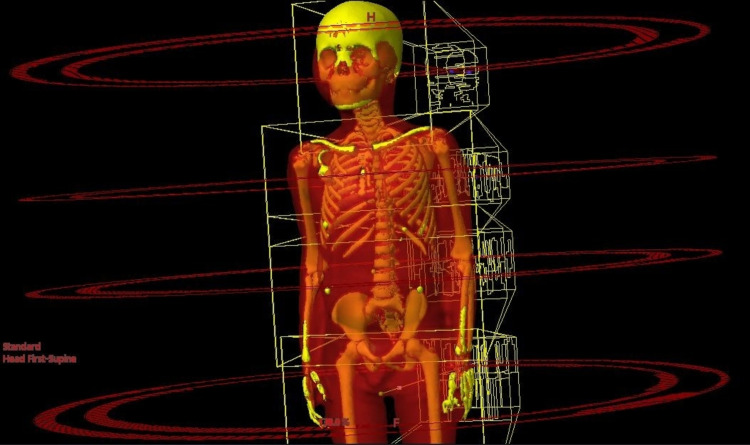
For both the techniques (conventional VMAT TBI and SIMBs), the same treatment fields were used, as seen above for a representative patient VMAT: volumetric modulated arc therapy; SIMBa: simultaneous integrated marrow and body approach; TBI: total body irradiation

To stay consistent with the conventional VMAT TBI plans for comparison, isocenters were placed in the same way. They were placed along the patient's longitudinal axis starting at the superior portion head. Isocenters were placed to ensure a minimum of 5 cm overlap in the AP-defined field size of each field. For each isocenter, one to three 6 MV VMAT arcs were used. To keep consistent with conventional VMAT TBI planning, the leg fields were planned with a modulated field-in-filed AP/PA technique and used as the base dose of the upper fields. It should be noted that in order to facilitate a planning comparison the arc orientations, parameters and isocenter locations were not adjusted from the clinically delivered TBI plan. For both the techniques - conventional VMAT TBI and SIMBa - the same treatment fields were used, as seen in Figure 2.

**Figure 3:**
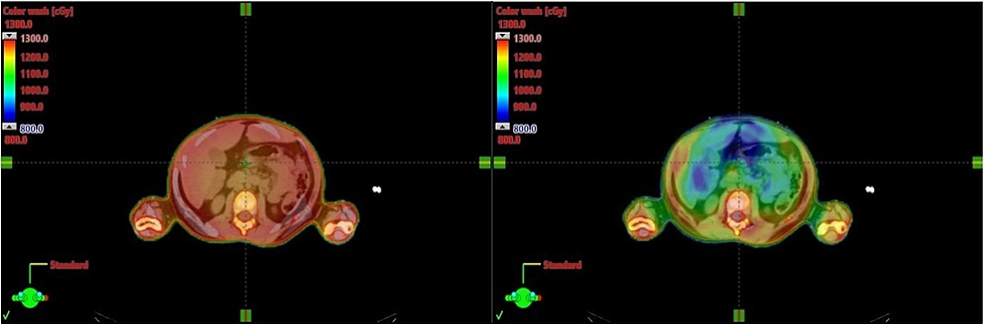
Representative axial slice of a conventional VMAT TBI (left) and SIMBa (right) prescribed to the same dose with the same color wash visualization The plans were normalized so that 100% of the PTV_Marrow received at least 90% of the dose with the PTV_TotalBody optimized to stay as close to 100% at 90% as possible. TBI: total body irradiation; VMAT: volumetric modulated arc therapy; SIMBa: simultaneous integrated marrow and body approach; PTV: planning target volume

The planning aims were to deliver a uniform dose of 12 Gy to at least 90% (V12 Gy[%] >= 90) of the PTV_Marrow and 8 Gy to at least 90% of the PTV_TotalBody while limiting the mean lung dose to less than 8 Gy. The plans were normalized so that 100% of the PTV_Marrow received at least 90% of the dose with the PTV_Totalbody optimized to stay as close to 100% at 90% as possible. Treatment plan evaluation was performed using dose-volume histogram (DVH) analysis. Organs at risk dose were assessed based on the mean dose to the organs. All reported doses to OARs are based on the contoured organ from the planning CT from the VMAT TBI with no changes. Planning and optimization for all fields and isocenters were done simultaneously. Table 2 shows the planning aim used during optimization and evaluation. Figure 3 shows a representative axial slice of a conventional VMAT TBI (left) and a SIMBa technique (right) prescribed to the same dose with the same color wash visualization. For statistical analysis, the statistical differences were evaluated using a paired sample t-test with a significance level of 0.05. 

**Table 2:** Conventional VMAT TBI and SIMBa planning aims VMAT: volumetric modulated arc therapy; SIMBa: simultaneous integrated marrow and body approach; TBI: total body irradiation

Name of Structure	Conventional VMAT TBI	SIMBa
PTV_Marrow	-	D90% >= 12 Gy
PTV_TotalBody	-	D90% >= 8 Gy
PTV_BodyEval	Max[Gy] < 16 ; D90% >= 12 Gy	Max [ Gy] < 16
_LungEval	Mean [Gy} < 8 Gy	Mean [ Gy] < 8
Spinal Cord	Max [Gy] < 15 @ 0.125cc	Max [Gy] < 15 @ 0.125cc
Oral Cavity	Max [Gy] < 15 @ 0.125cc	Max [Gy] < 15 @ 0.125cc
Bowel	Max [Gy] < 15 @ 0.125cc	Max [Gy] < 15 @ 0.125cc
Kidney (individual)	Mean [Gy] < 13	Mean [Gy] < 13
Whole Brain	Max [Gy] < 15 @ 0.125cc	Max [Gy] < 15 @ 0.125cc

## Results

**Figure 4:**
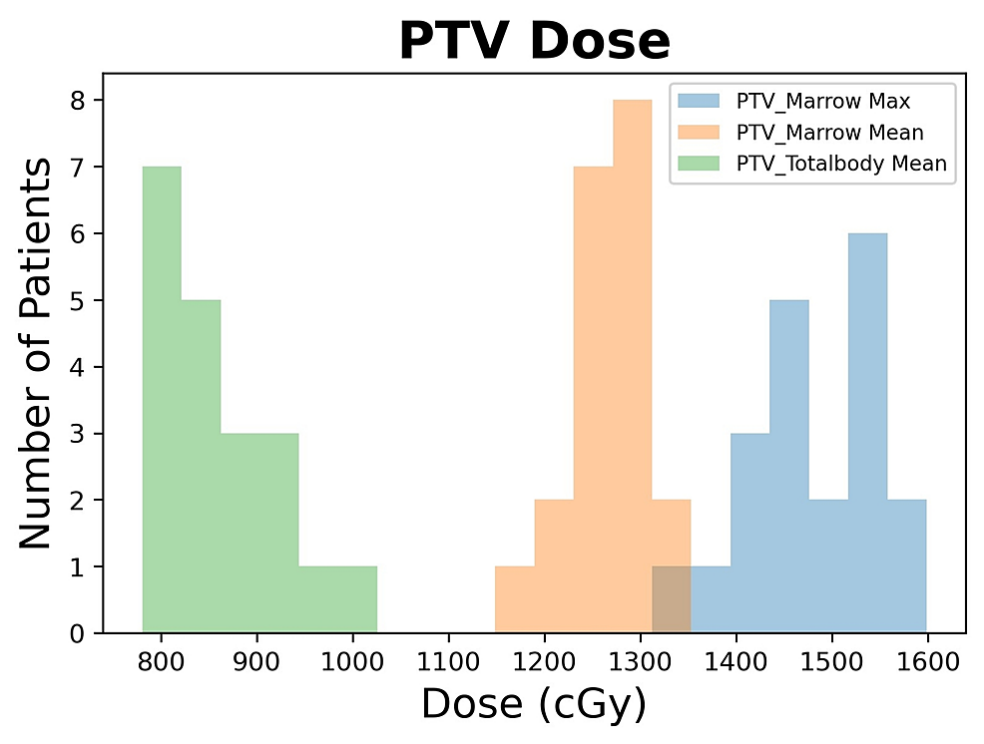
Histogram of the mean and max doses for PTV_Marrow and the mean for PTV_TotalBody for the SIMBa plans PTV: planning target volume; SIMBa: simultaneous integrated marrow and body approach

**Figure 5:**
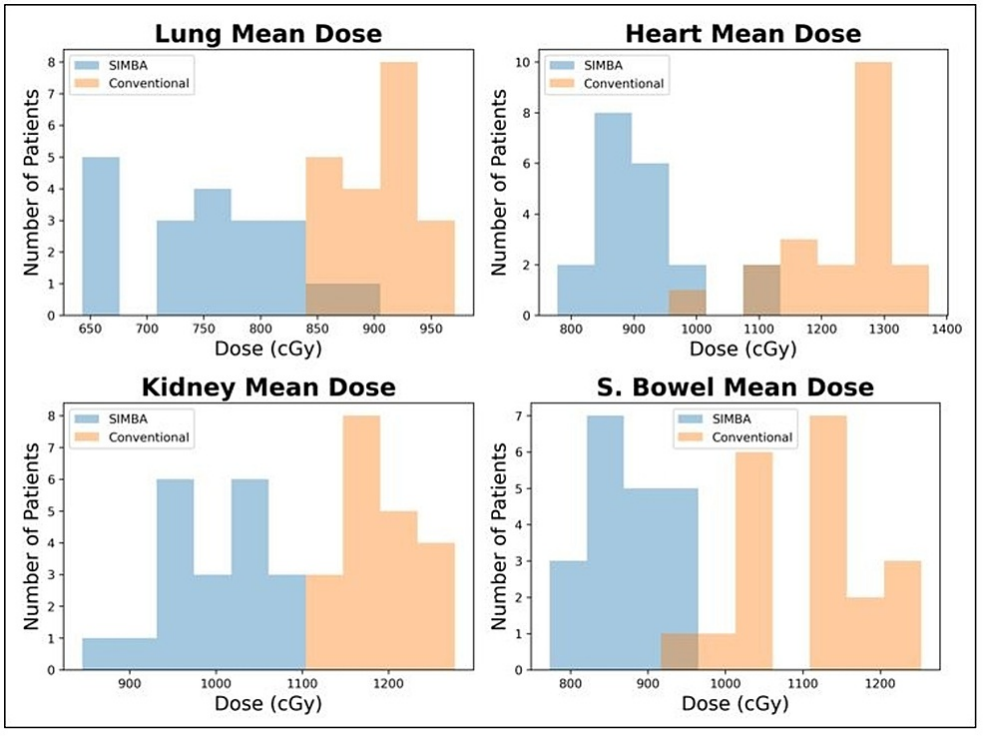
Comparison of the OAR mean dose between the conventional VMAT TBI and SIMBa techniques For each of the evaluated sites, the statistical differences were evaluated using a paired sample t-test with a significance level of 0.05. Each site was found to be significantly different with a maximum p-value of <0.008. VMAT: volumetric modulated arc therapy; SIMBa: simultaneous integrated marrow and body approach; TBI: total body irradiation; OAR: organ at risk

Using the SIMBa technique, all 20 patient plans achieved 12 Gy/8 Gy to at least 90% of the PTV_Marrow and PTV_TotalBody, respectively, with max doses of <16 Gy (130%). Table 3 shows the summary of the average mean dose values for selected OARs compared between conventional VMAT TBI and SIMBa. The coverage of the sanctuary sites was maintained despite a significant reduction to OARs. Figure 4 shows a histogram distribution of the mean/max doses for the PTV_Marrow and the PTV_TotalBody, and Figure 5 shows the histograms for the OAR mean doses.

**Table 3:** Summary of the average mean dose values for selected OARs compared between conventional VMAT TBI and SIMBa VMAT TBI OARs: organs at risk; SIMBa: simultaneous integrated marrow and body approach; VMAT: volumetric modulated arc therapy; TBI: total body irradiation; CNS: central nervous system

	Traditional VMAT TBI	SIMBa VMAT	Difference		Statistical Significance
	Average (cGy)	Average (cGy)	Average (%)	σ (%)	p-value
Bowel	1108	870	-21.3	4.2	<<0.05
Lung	902	756	-16.3	7.9	<<0.05
Heart	1233	916	-25.3	8.6	<<0.05
Kidney	1191	994	-16.4	6.2	<<0.05
CNS	1270	1229	-3.2	2.9	0.17

## Discussion

Figure 4 shows the mean and max dose distribution across all patients of the PTV_Marrow and the mean dose distribution across all patients of the PTV_TotalBody for the SIMBa technique. For all the cases except one, the PTV_Marrow mean value was at least 12 Gy, and for all cases, the PTV_Marrow max value was under 16 Gy. For the PTV_TotalBody, the mean value was at least 8 Gy for 16 of the 20 cases and at least 7.8 Gy for the remaining four cases. From this study, we were able to demonstrate that the SIMBa technique provides similar coverage to that of our conventional VMAT TBI PTVs and reduces dose to sensitive OARs. Additionally, coverage of the central nervous system (CNS), which served as our proxy for sanctuary sites, only saw a decrease of 3.2% in the mean dose value. The SIMBa technique produced observable differences in the organ dose for the lung, heart, kidney, and small bowel. Previous studies [23-29] have shown that, in general, decreases in dose in similar sites could result in clinically significant toxicities reductions, but future evaluation will be to be done to determine toxicity reduction for TBI specifically.

While SIMBa demonstrates similar target coverage and reduced dose to OARs in comparison to VMAT TBI, the current study still has several limitations. One of the limitations of this study is the number of patients available for analysis. Another limitation is that in order to facilitate a true comparison, isocenter locations, arc geometry, and plan parameters were not adjusted, as this was purely a planning comparison study. When this methodology is clinically implemented, changes to these variables may or may not provide an increased benefit to target coverage and OAR sparing and should be evaluated on a per-patient basis. The final limitation is that this study does not specifically address any potential improved radiobiological effects that might occur due to a reduction in dose. Without clinical evidence in the form of a structured clinical trial utilizing this technique, it is difficult to predict the impact that this treatment would have on toxicity and outcome for the patient. As the SIMBa technique evolves, future studies may focus more on these radiobiological factors.

Additionally, while SIMBa spares the OARs compared to VMAT TBI, there are some drawbacks of implementing the technique into routine clinical practice. Mainly, contouring the bone marrow can be extremely challenging and time-consuming. Therefore, it is often necessary to contour the entire skeletal system and use this system as a surrogate for bone marrow. Even this simplification, however, still results in increased planning time, which may make the process unappealing in a busy clinic or for a multicenter clinical trial. Auto-contouring software is developing at a rapid pace, and whole-body bone segmentation has already been implemented [30]. Popular methods include atlas-based methods [31] and convolutional neural networks [32], and both may also aid in the simultaneous contouring of other OARs including the kidneys, lungs, bowels, and others. In the future, these auto-contouring techniques will be explored and will be integrated into the SIMBa planning process to decrease the burden on the planning team and improve standardization across cases.

## Conclusions

This study demonstrates that SIMBa can dosimetrically provide the same PTV and sanctuary site doses as TBI while reducing the doses to OARs. When this methodology is clinically implemented, changes to these objectives may provide an increased benefit to target coverage and OAR sparing and should be evaluated on a per patient basis. It should also be noted that this study is intended to demonstrate the feasibility of planning using a SIMBa technique and specifically does not address any potential improved radiobiological effects that might occur due to a reduction in dose. The clinical effects should be evaluated in a structured clinical trial utilizing this technique. The future clinical trial will investigate the clinical benefit of these dosimetric advantages.
